# A Simulation-Based Study of the Effect of Brake Light Flashing Frequency on Driver Brake Behavior from the Perspective of Response Time

**DOI:** 10.3390/bs12090332

**Published:** 2022-09-14

**Authors:** Min-Chih Hsieh, Lan-Xin Chen, Yu-Chi Lee, Qin-Ming Liu

**Affiliations:** 1 Department of Industrial Engineering, University of Shanghai for Science and Technology, Shanghai 200093, China; 2 College of Management and Design, Ming Chi University of Technology, New Taipei City 243303, Taiwan

**Keywords:** driving behavior, rear-end collisions, flashing brake light, brake response time

## Abstract

To prevent vehicle crashes, studies have proposed the use of flashing signals (brake lights or other light indicators) to improve the driver’s response time when the leading vehicle is braking. However, there are no consistent results on the ideal flashing frequency of the brake lights. This study aimed to investigate different brake light flashing frequencies to assess their impact on braking response time. Twenty-four participants aged 25 to 30 were recruited. Two driving speed environments (50 and 80 km/h), three deceleration rates (0.25, 0.6, and 1 g), and four brake light flashing frequencies (0, 2, 4, and 7 Hz) were examined. Braking response time, average braking force, and braking response time ratio were used to evaluate the driving behavior. The results showed that the braking response time and average braking force were affected by the deceleration rate in the 50 km/h driving environment. In the 50 and 80 km/h driving environments, although there were no significant differences among the three deceleration rates, the braking response time decreased by 3–7% under the flashing brake light condition. These findings can be used as a reference for safety designs as well as future studies on driving behavior.

## 1. Introduction

Traffic accidents remain a significant cause of loss of life and property worldwide, and rear-end crashes account for the largest proportion. According to the annual report from the National Center for Statistics and Analysis [1], in 2019 rear-end collisions accounted for 32.5% of all crashes (2.194 million), 7.1% of fatal crashes (2346), 31.1% of crashes with injuries (0.595 million), and 33.2% of all property-damage-only crashes (1.597 million). In China, rear-end collisions are very common [2]. 

To prevent and reduce the number of rear-end collisions, further investigation into driver behavior is needed in conjunction with improvement in the traffic environment and the development of auxiliary devices on vehicles [3,4]. Most rear-end accidents are caused by several inappropriate behaviors of the following driver, such as distraction, inadequate perceptual discrimination, inappropriate interpretation of the traffic environment, and inadequate time-headway [5,6,7]. Previous studies have analyzed the causes of rear-end collisions by focusing on the driver’s braking behavior. They reported that the leading vehicle’s speed, deceleration rate, and headway distance can affect the braking response time of following drivers [7,8,9,10]. For instance, Schweitzer et al. [11] and Summala et al. [12] investigated the impact of the leading vehicle’s speed and the following vehicle’s distance on braking response time. They found that the following driver’s reaction time was faster when the following distance was shorter. Similar results were found by Aust et al. [13] and Engström [14]. 

In addition to speed and distance, the leading vehicle’s deceleration rate is a critical factor in rear-end collisions. Hulst et al. [7] investigated the influence of the leading vehicle’s deceleration rate on the following driver’s reaction time. Their results showed that the braking response time was longer when the deceleration rate was smaller; conversely, the greater the deceleration rate of the leading vehicle, the shorter the reaction time of the following driver. Wang et al. [15] investigated the relationship between reaction time and driver braking behavior under different levels of urgency, three rates of deceleration (0.3, 0.5, and 0.75 g), and two different headways (1.5 and 2.5 s). Their results showed that shorter distances could produce faster reaction times, and higher deceleration rates could lead to faster reaction times than lower rates. Such results confirm that the leading vehicle’s speed, deceleration rate, and distance significantly affect the braking judgment of the following driver. Therefore, improving the ability of the following driver to judge the dynamics of the leading vehicle is an important factor in reducing the number of rear-end collisions.

The brake light is a car component that enables drivers to judge the dynamics of the vehicles ahead of them. Although brake lights, as they function at present, indicate when a driver is pushing the brake pedal, they do not reflect how hard the driver is pushing [3,9]. Thus, following drivers have a difficult time judging the deceleration rate of the vehicle ahead of them via the brake light signal alone. They have to depend on other visual information, such as a change in the angular velocity during optical expansion, to confirm how quickly they are approaching the leading vehicle [9,16]. However, the change in the angular velocity of the leading vehicle might be insufficient to allow the following driver to judge the deceleration rate correctly [3]. Hence, other studies have focused on ways to improve the design of the brake lights to include additional information that can enable following drivers to determine the deceleration rate of the leading vehicle [17,18,19,20]. 

From the perspective of visual characteristics, visual attention is easily attracted by items that are bright, colorful, and changeable, and these characteristics can be used to design visual warning systems [21]. Wierwille et al. [18] compared the conventional brake light and a flashing warning light at a 4 Hz flashing frequency on the following driver’s braking response time. Their results showed no significant difference between a conventional brake light and a flashing warning light on braking response time, although the following driver’s brake reaction time was reduced from 0.25 s to 0.35 s in the flashing warning light condition. Similar results have been found by Li et al. [19]. They compared following drivers’ braking response times with a conventional brake light and a flashing brake light. In their study, brake lights flashed at 3.6 Hz when the deceleration of the vehicle was 0.6 g. Their results showed no significant difference between the conventional brake light and flashing brake light on brake response time, although the following driver’s braking response time was reduced by 0.14 s in the flashing brake light condition. In the study cited above, flashing light systems with low frequencies (3.6 and 4 Hz) were insufficient to reduce the reaction time of the following drivers significantly. By contrast, Sohrabi [20] found a significant difference in the braking reaction time of the following driver between a conventional brake light and a brake light at a 7 Hz flashing frequency, with the braking response time for the flashing brake light significantly faster than that for the conventional brake light. 

However, this does not mean that the flashing brake light at a high frequency (7 Hz) significantly improved the response time of the participants relative to a brake light at a low frequency (3.6 and 4 Hz), which can be discussed from several viewpoints. First, from the perspective of vehicle speed, Wierwille et al. [18] and Sohrabi [20] did not set the vehicle speed as an independent variable in their experiment. By contrast, Li et al. [19] applied three vehicle speeds in their study: 60, 80, and 100 km/h. Second, from the perspective of deceleration rate, Wierwille et al. [18] and Sohrabi [20] did not consider the effect of the deceleration rate of the leading vehicle on the response time of the following driver, whereas Li et al. [19] used three deceleration rates (0.4 g, 0.6 g, and 0.8 g) to simulate a situation that would be encountered in real-world driving environments. 

Finally, from the perspective of brake light flashing frequency, each of these three studies [18,19,20] used only one flashing frequency to assess the brake response time of the following driver. From these three points of view, it can be seen that Wierwille et al. [18] and Sohrabi [20] considered fewer factors in their experiment, which limits the application of their results. Furthermore, although Li et al. [19] added independent variables for vehicle speed and deceleration rate, they used only one brake light flashing frequency (3.6 Hz) in their experiments, and therefore their findings could not explain the effect of higher or lower brake light flashing frequencies on the braking response time of following drivers. Moreover, although visual attention is easily attracted by flashing signals, the effect of different flashing frequencies on the response time is unclear. Therefore, further research is needed to understand the effect of different brake light flashing frequencies on the braking response time of following drivers considering different deceleration rates and the speed of the leading vehicle.

Considering the issues elucidated above and to address these gaps in the literature, this study investigated the effect of speed, deceleration rate, and brake light flashing frequency of the leading vehicle on the braking response time of the following driver. It is anticipated that the results of this study could be used as a reference for brake light design, which in turn could reduce the occurrence of rear-end accidents and improve driving safety.

## 2. Methods

### 2.1. Participants

This study recruited 24 participants (aged 25 to 30; 14 males and 10 females) who participated voluntarily in our experiment. The average age of the participants was 26.3 years, and each had at least five years and 60,000 km (over 50 km per work day) driving experience. The inclusion criteria of participants in this experiment were: (1) A valid C1 or C2 driving license for China, (2) normal or corrected-to-normal vision, (3) no anomalous color vision, and (4) absence of psychiatric and sleep disorders.

### 2.2. Driving Simulator 

**Figure 1:**
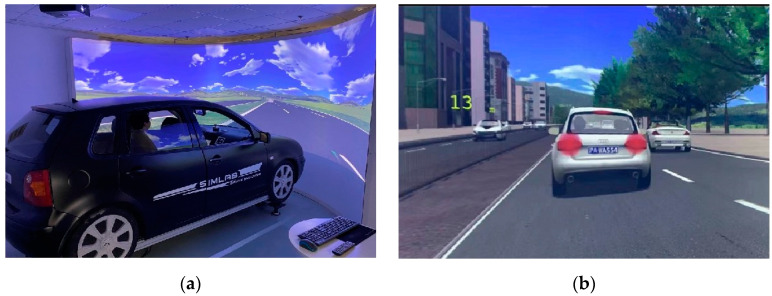
(**a**) The SILAB driving simulator and (**b**) the simulated ordinary road driving environment.

It is difficult to collect data such as vehicle speed, brake pedal force, and deceleration rate safely and efficiently in real-world driving environments [22,23]. Consequently, to achieve our objective, we used a driving simulator that is already widely applied in studies related to driving simulation [24,25,26]. Specifically, we used the SILAB driving simulator (WIVW GmbH, Veitshöchheim, Germany) to construct the driving environment for our experiment, located at INFO. instruments in Shanghai. We simulated the cockpit of a VW Polo vehicle with four real systems: steering simulation, brake and acceleration pedals, instrument control interface, and noise and vibration simulation systems. As such, the controls were those of an ordinary automatic vehicle. In addition, a 210-degree curved projection display (height: 2.62 m; radius: 2.8 m) presented the driving environment, and the distance from the participant to the display was 2.8 m. The driving environments were a 20-km long ordinary road and a 50-km long viaduct. The leading vehicle drove ahead at a speed of approximately 50 km/h on the ordinary road (80 km/h on the viaduct), passing was not allowed, and there were no intersections. The lanes on the ordinary road had a width of 3.25 m (3.5 m on the viaduct). On level and straightaways, any impact of horizontal bends and longitudinal slopes on the braking and steering of the drivers was removed. In addition, the field of view was wider and there were fewer buildings in the viaduct environment than in the ordinary road environment. The SILAB driving simulator and the ordinary road driving environment are shown in Figure 1. 

### 2.3. Experimental Environment and Design

We used two types of roads in Shanghai, ordinary roads and viaducts, to gain a complete picture of the response time of following drivers to the speed, deceleration rate, and flashing brake lights of the leading vehicle. Both the ordinary road and viaduct in the experiment had six lanes in two directions. An Audi A4 sedan, 4.4 m long and 2.05 m wide, was the leading vehicle in the experiment. The participants needed to follow the leading vehicle and maintain a safe distance (*SD*) from it. The *SD* was based on the road’s speed limit. According to the Shanghai traffic laws, the speed limit is 50 km/h on an ordinary road and 80 km/h on a viaduct, with SDs on an ordinary road and a viaduct of 13 m and 32 m, respectively. The calculation equation for *SD* is as follows:

(1) $$SD=\frac{V^{2}}{2\times g\times \mu }$$

where *g* = 9.8 m/s^2^ and μ = 0.8 (coefficient of friction).

With regard to deceleration rate, previous studies have indicated that different deceleration rates affect the braking response time of following drivers [15,19]. Wood and Zhang [27] reported that the minimum and maximum deceleration rates among 2971 natural drivers were 0.23 g and 1.09 g, respectively. These represent the minimum and maximum deceleration rates that a driver may experience during normal daily driving. In addition, a deceleration of 0.6 g is generally assumed as the threshold for an emergency braking situation [28,29]. Notably, Li et al. [19] used 0.6 g as the deceleration rate for emergency braking to trigger the flashing brake/hazard system. Considering the tuning of the driving simulator used in this study, we therefore utilized deceleration rates of 0.25 g, 0.6 g, and 1 g to present the braking situation of the leading vehicle and to measure the braking response time of the participants in our experiment. 

Previous studies that have examined the flashing frequency of brake lights investigated frequencies of 3.6, 4, and 7 Hz [18,19,20], with mixed outcomes. To verify the effectiveness of the flashing brake light, flashing frequencies of 4 and 7 Hz were used in this study. In addition, a low-frequency flashing of 2 Hz and a traditional brake light (0 Hz) were applied as extra factors in the experiment to provide a more comprehensive analysis of the braking response time of following drivers. Moreover, in addition to the effect of brake light frequency on braking response time, the speed and deceleration rate of the leading vehicle should be considered.

In light of the above, we investigated three independent variables: driving environment (road speeds), deceleration rate, and flashing frequency of the brake lights. We established two driving environments based on differing speed limits of 50 km/h (ordinary roads) and 80 km/h (viaducts). For the deceleration rate, we set three distinct levels: 0.25, 0.6, and 1 g. We set the flashing frequencies at 0 (conventional brake light), 2, 4, and 7 Hz because these were the critical flashing brake light factors. Accordingly, we employed a balanced factorial design with 2 (driving environment) × 3 (deceleration rate) × 4 (flashing frequency) combinations. All were within-subject factors.

### 2.4. Experimental Scenario and Procedure

Before the formal experiment began, each participant was shown how to use the driving simulator and allowed at least ten minutes to become familiar with the characteristics of the simulation system, such as the accelerator pedal, brake pedal, steering wheel, driver’s seat, and the two driving environments, to minimize the effect of extraneous factors on the results of this experiment. If dizziness occurred during the experiment, the subject was withdrawn from participation. All participants signed informed consent forms. 

As stated, we used two driving speed environments: 50 km/h (ordinary road) and 80 km/h (viaduct). The participants were randomly assigned to these two driving environments. Half of the participants began the formal experiment in the 50 km/h driving environment, while the other half began in the 80 km/h driving environment; these were then reversed in the second half of the experiment. The participants controlled the car’s speed and brake while following the leading vehicle at a requested distance (13 m in the 50 km/h driving environment and 32 m in the 80 km/h driving environment) on a straight road during the experiment, and they reacted to the leading vehicle’s braking in the same manner as in a real-world driving situation. In other words, the main task in the experiment was that of deceleration; when the brake light of the leading vehicle came on, the participants needed to determine whether there was a risk of collision and adjust the speed of their vehicle through the brake pedal to avoid a collision as soon as possible. The secondary task was to follow the leading vehicle and maintain a specific safety distance. The distance between the leading and following vehicles was displayed in real time on the left side of the driver’s field of vision (Figure 1b). In addition, a “speed up” or “slow down” message was displayed near the real-time distance when the following vehicle was not within the specified distance.

**Figure 2:**
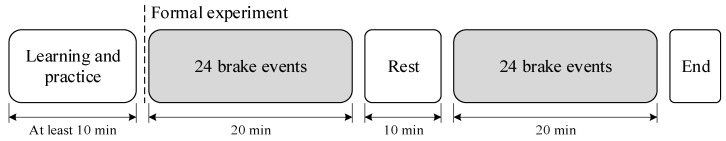
The experimental process.

There were twelve braking events randomly distributed in each driving environment, with each driving environment taking roughly 20 min to complete these events. The average interval of each braking event followed a normal distribution, with a mean of 1.2 min and a standard deviation of 0.6 min. After the twelve braking events, the participants were asked to step away from the vehicle and rest for 10 min before the next driving test. To immerse the participants in the experiment, the driving simulator provided engine noises and inertial forces as the vehicle accelerated or decelerated, engendering a more realistic driving experience. The experimental process is shown in Figure 2. 

### 2.5. Data and Statistical Analysis

**Figure 3:**
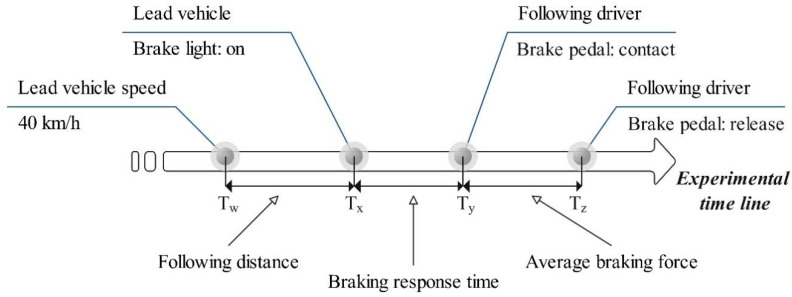
The relationship among braking response time, average braking force, and average following distance.

During the experiment, we calculated the influence of the flashing brake light under the different driving conditions on the following driver’s braking response time, defined as the interval between the activation of the leading vehicle’s brake light and the initial foot contact of the following driver with the brake pedal [19]. We assumed T_x_ as the time when the leading vehicle’s brake light turned on and T_y_ as the time when the following driver’s foot initially contacted the brake. Thus, the braking response time (RT) was calculated as the difference between T_x_ and T_y_. In addition, we calculated the ratio of the braking response time to each flashing brake light frequency to the response time to conventional brake lights to assess the efficiency of the flashing brake lights in improving the braking response time. We assumed T_z_ as the time when the following driver released the brake pedal the first time after T_y_, while the average braking force was denoted as the force of the following driver depressing the brake pedal from T_y_ to T_z_. We recorded the average following distance between the leading and following vehicles to evaluate the braking response time associated with the speed of the leading vehicle, the deceleration rate, and the flashing frequency. The relationship among T_x_, T_y_, and T_z_ is shown in Figure 3. The calculations of braking response time, deceleration rate, average braking force, and average following distance are as follows.

(2) $$Brake\;response\;time=T_{y}-T_{x}\;\;$$

(3) $$Brake\;response\;time\;ratio=\left(1-\frac{R_{i}}{R_{c}}\right)\times 100\%\;$$

where $R_{c}$ denotes the response time in the conventional brake light condition and $R_{i}$ denotes the response time in the brake light conditions with *i* Hz flashing frequency, where i=2, 4,or 7.

(4) $$Average\;braking\;force=\frac{\sum_{i}\left(F_{T_{y}}+F_{T_{y}+i}+F_{T_{y}+2i}+\cdots +F_{T_{z}}\right)}{T_{z}-T_{y}}$$

where $F_{T_{y}}$ and $F_{T_{z}}$ denote the different braking forces the following driver applied to the brake pedal at $T_{y}$ and $T_{z}$, respectively, and *i* denotes the time interval of the driving system sampling.

(5) $$Average\;following\;distance=\frac{\sum_{i}\left(D_{T_{w}}+D_{T_{w}+i}+D_{T_{w}+2i}\cdots +D_{T_{x}}\right)}{T_{x}-T_{w}}\;$$

where $D_{T_{x}}$ and $D_{T_{w}}$ denote the real-time distances between the leading and following vehicles at $T_{x}$ and $T_{w}$, respectively, and *i* denotes the time interval of the driving system sampling.

We used the Kolmogorov–Smirnov test for our data analysis to evaluate the normality of the data, the Levene’s test for homogeneity of the variance, and analysis of variance (ANOVA) and post hoc analysis to verify the difference in the braking response time and average braking force under the different brake light flashing frequencies and deceleration rates in the 50 and 80 km/h driving environments. *p*-values were applied to evaluate the significance (*p* < 0.05) for each statistical test. We used SPSS 16 to perform all our statistical analyses.

## 3. Results

### 3.1. Braking Response Time

Generally, the ANOVA results in terms of significance level (*p*-values) and effect size (η^2^) showed that the driving environment (F = 97.319, *p* < 0.001, η^2^ = 0.162) and deceleration rate (F = 4.888, *p* = 0.008, η^2^ = 0.019) significantly affected braking response time and that there was an interaction among driving environment, deceleration rate, and flashing frequency (F = 2.598, *p* = 0.017, η^2^ = 0.017).

In addition, we separated the main effects of the braking response time into the two driving environments and analyzed them in terms of significance level (*p*-values) and effect size (η^2^). In the 50 km/h driving environment, the results show that while the deceleration rate significantly affected the braking response time (F = 5.388, *p* < 0.05, η^2^ = 0.041), there was no significant effect from the flashing frequency or from the interaction between the deceleration rate and flashing frequency on braking response time (Table 1).

**Figure 4:**
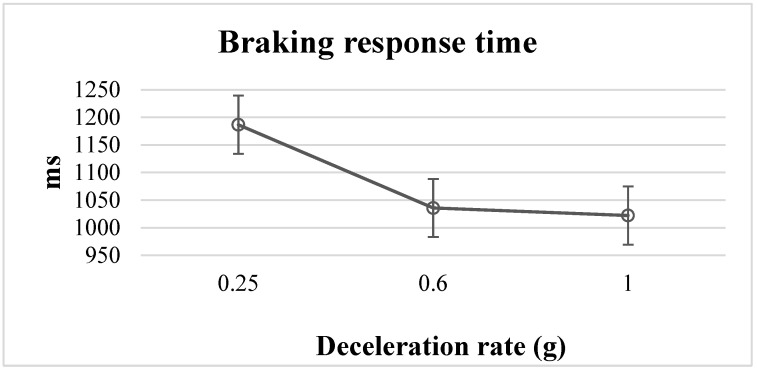
Braking response time for three deceleration rates in the 50 km/h driving environment.

In the post hoc analysis results, there were significant differences between the deceleration rates of 0.25 g and 0.6 g (*p* < 0.05) and the deceleration rates of 0.25 g and 1 g (*p* < 0.05) on braking response times (Figure 4). This confirmed that the braking response times for the events with the 0.6 g and 1 g deceleration rates were significantly faster than that for the 0.25 g deceleration rate. In the 80 km/h driving environment, there was no significant effect from the deceleration rates, the flashing frequencies, or their interactions on braking response time.

### 3.2. Average Braking Force

**Figure 5:**
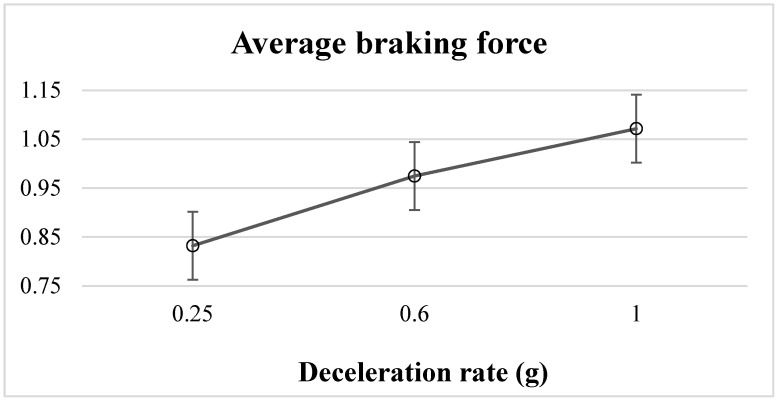
Braking response force for three deceleration rates in the 50 km/h driving environment.

The ANOVA results in terms of significance level (*p*-values) and effect size (η^2^) showed that the driving environment (F = 45.197, *p* < 0.001, η^2^ = 0.082) and deceleration rate (F = 4.812, *p* = 0.009, η^2^ = 0.019) significantly affected the average braking force. The main effects of the average braking force were separated into the two driving environments and analyzed in terms of significance level (*p*-values) and effect size (η^2^). In the 50 km/h driving environment, the results indicated that while the deceleration rate of the leading vehicle had a significant effect on the average braking force (F = 4.229, *p* < 0.05, η^2^ = 0.032), there were no significant effects from the flashing frequencies or the interaction between the deceleration rate and flashing frequency on average braking force. Moreover, the results of the post hoc analysis showed that the average braking force in the brake event of the 1 g deceleration rate was significantly higher than that of the 0.25 g deceleration rate (*p* = 0.011) (Figure 5). With regard to the 80 km/h driving environment, there were no significant effects in terms of deceleration rate, flashing frequency, or their interaction on the average braking force.

### 3.3. Average Following Distance

In the 50 km/h driving environment, no significant effects were found in terms of the deceleration rate (F = 0.599, *p* > 0.05), flashing frequency (F = 1.138, *p* > 0.05), or their interaction (F = 2.635, *p* > 0.05) on the average following distance. We found the same results in the 80 km/h driving environment, where there were no significant effects from the deceleration rate (F = 0.896, *p* > 0.05), flashing frequency (F = 1.810, *p* > 0.05), or the interaction effect (F = 3.142, *p* > 0.05) on the average following distance.

### 3.4. Braking Response Time Ratio

The effect of the deceleration rate on the braking response time ratio in the two driving environments is summarized in terms of significance level (*p*-value) and effect size (η^2^) in Table 2. In the 50 km/h driving environment, the results showed that the braking response time at 2 Hz flashing frequency was 1.81% lower than that at the 0 Hz flashing frequency. The braking response time at 4 Hz flashing frequency was 3.66% higher than that at 0 Hz flashing frequency, and the braking response time at 7 Hz flashing frequency was 7.7% higher than that at 0 Hz flashing frequency. However, we found no significant effect between the deceleration rate and the braking response time ratios at 2 Hz to 0 Hz, 4 Hz to 0 Hz, or 7 Hz to 0 Hz.

In the 80 km/h driving environment, the results showed that the braking response time at 2 Hz flashing frequency was 4.838% higher than that at 0 Hz flashing frequency, the braking response time at 4 Hz flashing frequency was 5.305% higher than that at 0 Hz flashing frequency, and the braking response time at 7 Hz flashing frequency was 7.546% higher than that at 0 Hz flashing frequency. Here, we again found no significant effect between the deceleration rate and the brake response time ratio at 2 to 0 Hz, 4 to 0 Hz, or 7 to 0 Hz.

## 4. Discussion

Our objective was to investigate the effects of the driving environment, deceleration rate, and brake light flashing frequency on the following driver’s braking response time, average braking force, and average following distance. The results showed that the speed and deceleration rate significantly affected the braking response time and average braking force. The braking response times at the 0.6 g and 1 g deceleration rates in the 50 km/h driving environment were significantly faster than that at the 0.25 g deceleration rate, and the average braking force at the 1 g deceleration rate in the 50 km/h driving environment was significantly stronger than that at the 0.25 g deceleration rate.

### 4.1. Average Braking Force and Following Distance

As stated above, we found that the deceleration rate had a significant effect on the braking force in the 50 km/h driving environment and that the greater the deceleration rate, the stronger the braking force. This indicates that when the relative speed of two vehicles increases, the following driver adopts emergency braking to avoid a collision. However, there was no significant effect between the deceleration rate and average braking force in the 80 km/h driving environment. Considering the following distance between the leading and following vehicles, the results showed that the following distance was not affected by the flashing frequency of the brake light or the deceleration rate in either the 50 or 80 km/h driving environment. The implication is that the participants maintained a strong safety distance during the task. 

According to the drivers’ vision characteristics, when two vehicles have moved the same distance, the closer vehicle will be perceived to be at a greater distance than the farther vehicle [21]. In this experiment, 13 m and 32 m were the safety following distances in the 50 and 80 km/h driving environments, respectively. Therefore, it is possible that the participants were more susceptible to this visual stimulus in the 50 km/h driving environment, which in turn inspired a stronger braking force to avoid a collision at a high deceleration rate. In the 80 km/h driving environment, however, the distance between the leading and following vehicles was farther than that in the 50 km/h driving environment, and the participants might have been exposed to less visual stimulus, meaning that the braking force of the following driver was not affected by the deceleration rate of the leading vehicle.

### 4.2. Brake Response Time

Our results for braking response times in the 50 km/h driving environment were similar to those in previous studies, in that the deceleration rate of the leading vehicle significantly affected braking response times [9,10]. However, we found no significant effect between the braking response time and the deceleration rates of 0.6 g and 1 g. In addition, we found no significant effect between deceleration rates and braking response times in the 80 km/h driving environment, which differs from the results in previous studies. One possible reason for this may be that the relative approach speed between the leading vehicle and the following vehicle was too fast, exceeding the threshold for visual recognition of angular velocity change. In this experiment, the lowest relative approach speed between the leading and following vehicles was in the 50 km/h driving environment at a 0.25 g deceleration rate to enable observation of the longest braking response time, which was significantly different from the other experimental conditions. Similar results were reported by Wang et al. [15], where the braking response time at the 0.3 g deceleration rate was significantly lower than that at the 0.5 and 0.7 g deceleration rates. Although the driving speed environment in Wang et al.’s study was at 120 km/h, which differs from the speeds used in our study, they found a phenomenon similar to the threshold for visual recognition of angular velocity change. One of the reasons for these different findings may be differences in our research objectives, experimental methods, and the equipment used, although the threshold for visual recognition of angular velocity change has been infrequently referenced in previous studies. Thus, the relationship between vision and angular velocity needs to be further investigated.

With regard to brake light flashing frequency, our results showed no significant difference between flashing frequency and braking response time in the two driving environments. The implication is that the flashing brake light did not motivate a faster response time among our participants. We calculated the braking response time ratio of the flashing brake light frequencies at 2, 4, and 7 Hz to the reaction time of the brake lights at 0 Hz (conventional brake lights). The results showed that except for the braking response time at 2 Hz flashing frequency in the 50 km/h driving environment, where the response time increased by 1.8% compared with the response time to the conventional brake light, the response times decreased by 3–7% in all other conditions (Table 2). This means that the flashing brake light had the effect of motivating the participants to respond faster.

Previous studies have reported that current brake light systems do not reflect how hard the leading driver is braking [3,9], and the following drivers have to depend on other visual information to confirm the status of the leading vehicles [9,16]. Thus, studies such as the present one have begun to focus on brake light design and the use of flashing signals as ways to add more visual cues to brake light action. 

From a statistical point of view, however, we found no significant effect between the braking response time ratio and deceleration rate under our two different driving environments. Several of our results are similar to those in previous studies. In previous related studies, such as Wierwille et al. [18] and Li et al. [19], although low-frequency flashing brake lights (e.g., 3.6 and 4 Hz) reduced the braking response time, there was no significant difference compared with conventional brake lights. This held true in our study as well.

Compared to the fixed deceleration rate (0.6 g) used in the study by Li et al. [19], we went further by adding different deceleration rates (0.25 g and 1.0 g) for the leading vehicles in order to investigate the relationship between these different rates and the flashing frequency of the brake light; however, we found that the braking response time was not affected by these factors. Thus, we can speculate that a flashing brake light at a frequency of 4 Hz or less does not affect the braking response time of the following driver. However, under the experimental condition where the brake lights were flashing at a frequency of 7 Hz, although the reaction time was about 7.5% faster than that for conventional brake lights (Table 2), there was no significant difference between the reaction time and deceleration rate; this result is inconsistent with that in the findings of Sohrabi [20], which indicated that brake lights with a flashing frequency of 7 Hz could significantly reduce the braking response time of following drivers. However, Sohrabi did not elaborate on the details of the experiment in his study, such as following distance, driving environment, or deceleration rate of the leading vehicle, making it difficult to compare his results with ours. Considering the above discussion, we can see that the braking response time is not easily affected by the brake light at a 7 Hz flashing frequency.

Although visual attention is easily attracted to items that are bright, colorful, and changeable [21], these advantages, when applied to brake lights, did not have an impact in our study or in those conducted in the past. The reason for this may be related to the driving environment. In a real driving environment, drivers need to pay attention to both the vehicle in front and to other conditions such as traffic signals and road signs. Although the brightness principle is behind the design of traditional brake lights and the flashing mode changes the light from bright to changeable, from a visual attention point of view, both bright and changeable characteristics attract visual attention; thus, whether there is a trade-off or an additive effect between these two principles needs to be studied in greater depth.

### 4.3. Research Limitations

Several limitations of our study warrant discussion. First, although driving simulation systems are considered an efficient and safe means of allowing the simulation of high-risk collisions that occur in real-world driving environments, the question of how to elicit similar driving behaviors when using driving simulation systems as a research tool as in the real-world driving environments is an ongoing research topic [22,30,31]. Thus, the results we obtained might not fully reflect the situation of a true driving environment. This means that the effectiveness of flashing brake lights needs to be further verified in a real-word environment. Second, this study only added the flashing mode on the traditional brake light, rather than any additional visual cues. Thus, the results only demonstrate that the brake light designed to flash had no effect on the braking response time of the participants. Third, we considered only two speeds (50 and 80 km/h) in the relationship between the flashing brake lights and response time, which means that higher and lower following speeds need to be investigated further. Fourth, participants with different backgrounds (sex, education, age, vocation, driving style, etc.) might have influenced the results of this experiment. For instance, taxi drivers are known to frequently commit speeding offenses [32]. However, this study did not collect and analyze any background information of the participants or investigate driving behavior for the specific groups. Consequently, a certain level of bias may exist in the results of this study. Finally, according to the braking system type (disc brake or drum brake), the efficiency of brakes can differ, which in turn affects the braking distances of the vehicle. However, the driving simulator used in this study does not provide a module for different braking systems for the user to select. Therefore, it is unknown how different braking systems could affect the braking distance in this study.

## 5. Conclusions

In this study, we investigated the effect of four flashing brake light frequencies, three deceleration rates, and two following speeds on braking response times. To this end, we used the ratio of braking response time to evaluate the ratio of the reduction in response times under different brake light flashing frequency conditions. The results showed that the braking response in a 50 km/h driving environment was affected by the deceleration rate, whereas that in an 80 km/h driving environment was not. Furthermore, the flashing frequencies in the two driving environments did not affect the braking response times. 

Visual attention is easily attracted to changeable items, and we considered this in our evaluation of the effect of flashing brake lights on braking response time. Although the experimental purpose, process, and methodology differed from those of previous studies, our results were similar in general; our main finding is that brake lights below a 7 Hz flashing frequency do not affect the braking response time of the following driver. Compared with conventional brake lights, however, flashing brake lights reduced the braking response time by 3–7% under different conditions, such as the speed and deceleration rate of the following vehicle, meaning that while flashing brake lights could reduce the brake response time, the effect was not obvious.

Currently, vehicles with autonomous driving system are not widely used, and people need to control vehicles based on visual cues in real-word driving environments. The brake light is one such visual cue, and has an important impact on the deceleration of the vehicle. It affects both the speed and flow of traffic, and may cause rear-end collisions and other traffic conflicts [33,34,35]. Therefore, in order to further understand the impact of flashing brake lights on driving safety, further exploration of the issue in several research directions considering the limitations of this study is necessary. First, the effect of brake lights with a flashing frequency higher than 7 Hz on the driver’s braking response time; second, a higher or lower vehicle speed; third, the manner in which the flashing signal is displayed (co-displayed with the brake light or an additional display system); and fourth, an interaction analysis of the participants’ background and driving behavior, which might influence the results of braking response time. Finally, considering the visible differences between the simulated and real driving environments, it is important to verify that the experimental data from these two driving environments is consistent. It is anticipated that our study will lead to a deeper understanding of the behavior and responses of drivers in different driving environments, and that the driving environment will be redesigned and improved in accordance with the results, thereby improving traffic safety.

## Figures and Tables

**Table 1:** ANOVA results: Braking response time and average braking force in the two driving environments.

Braking Response Time						
	**50 km/h**			**80 km/h**		
**Source**	**F**	***p*** **-Value**	**η** ^**2**^	**F**	***p*** **-Value**	**η** ^**2**^
**Deceleration rate (D)**	5.388	0.005 *	0.041	1.077	0.342	0.008
**Flashing frequency (F)**	1.078	0.359	0.013	1.276	0.283	0.015
**D × F**	0.831	0.547	0.019	1.978	0.069	0.045
**Average Braking Force**						
	**50 km/h**			**80 km/h**		
**Source**	**F**	***p*** **-Value**	**η** ^**2**^	**F**	***p*** **-Value**	**η** ^**2**^
**Deceleration rate (D)**	4.229	0.016 *	0.032	1.673	0.190	0.013
**Flashing frequency (F)**	0.092	0.964	0.001	1.755	0.156	0.020
**D × F**	0.547	0.772	0.013	0.624	0.711	0.015

*: *p* < 0.05.

**Table 2:** ANOVA results for the braking response time ratio in the two driving environments considering the deceleration rate.

50 km/h	Deceleration Rate				
Brake Response Time Ratio	Mean (%)	S.D. (%)	F	*p*-Value	η^2^
**2 Hz/0 Hz**	−1.810	33.028	2.333	0.105	0.069
**4 Hz/0 Hz**	3.662	27.603	1.279	0.286	0.039
**7 Hz/0 Hz**	7.706	24.638	1.290	0.282	0.039
**80 km/h**	**Deceleration Rate**				
**Brake Response Time Ratio**	**Mean (%)**	**S.D. (%)**	**F**	***p*** **-Value**	**η** ^**2**^
**2 Hz/0 Hz**	4.838	44.558	1.944	0.152	0.058
**4 Hz/0 Hz**	5.305	40.984	2.555	0.086	0.075
**7 Hz/0 Hz**	7.546	37.741	2.425	0.097	0.071

S.D.: Standard deviation.

## Data Availability

The datasets generated and analyzed during the current study cannot be made publicly available owing to privacy and confidentiality reasons.
